# Impact of appropriateness in clinical practice: data from a single-centre nuclear cardiology laboratory

**DOI:** 10.1093/ehjimp/qyad036

**Published:** 2023-11-14

**Authors:** Riccardo Liga, Dario Grassini, Assuero Giorgetti, Enrico Grasso, Stefano Dalmiani, Alessia Gimelli

**Affiliations:** Università di Pisa, Lungarno Pacinotti 43 56126 Pisa, Italy; Division of Cardiology, Cardiothoracic and Vascular Department, Azienda Ospedaliero-Universitaria Pisana, Pisa, Italy; Università di Pisa, Lungarno Pacinotti 43 56126 Pisa, Italy; Division of Cardiology, Cardiothoracic and Vascular Department, Azienda Ospedaliero-Universitaria Pisana, Pisa, Italy; Imaging Department, Fondazione Toscana Gabriele Monasterio, Via Moruzzi 1, Pisa, Italy; Imaging Department, Fondazione Toscana Gabriele Monasterio, Via Moruzzi 1, Pisa, Italy; Imaging Department, Fondazione Toscana Gabriele Monasterio, Via Moruzzi 1, Pisa, Italy; Imaging Department, Fondazione Toscana Gabriele Monasterio, Via Moruzzi 1, Pisa, Italy

**Keywords:** myocardial scintigraphy, appropriateness, guideline adherence, myocardial ischaemia

## Abstract

**Aims:**

To verify the level of appropriateness of referral to our nuclear cardiology laboratory for stress myocardial perfusion imaging (MPI) and explore the correlation between test appropriateness patterns and ischaemia.

**Methods and results:**

In 1870 consecutive patients (mean age 73 ± 12 years; 33% female) undergoing MPI, the level of imaging test appropriateness was evaluated according to the 2023 Appropriate Use Criteria (AUC) and the current European Society of Cardiology (ESC) guidelines for the management of chronic coronary syndromes. The evidence of moderate-to-severe ischaemia (i.e. summed difference score >7) was recorded. According to the AUC criteria, the MPI of 1638 (88%), 130 (7%), and 102 (5%) patients could be classified as ‘appropriate’, ‘inappropriate’, and ‘uncertain’, respectively. Similarly, in 1685 (90%) patients, the referral to MPI was adherent to ESC guidelines, while in 185 (10%), it was not. The majority of appropriate MPI tests showed the presence of moderate-to-severe ischaemia (55%), while only a limited number (10%; *P* < 0.05) of MPI tests with uncertain clinical appropriateness or clearly inappropriate indications did not. In patients managed adherently to ESC guidelines, invasive coronary angiography more frequently showed obstructive coronary artery disease (CAD) (93 vs. 47%, *P* < 0.001) and led to coronary revascularization (65 vs. 23%, *P* < 0.001) compared with patients managed non-adherently.

**Conclusion:**

In a single-centre, single-national, single-modality population, the current rate of appropriate MPI tests is high. Appropriate referrals are associated with a higher probability of moderate-to-severe ischaemia and better downstream resource utilization than inappropriate ones.

## Introduction

In recent years, there has been a marked surge in the adoption of cardiovascular imaging techniques, leading to questions about their economic sustainability. This concern is particularly pertinent to imaging techniques that entail potential collateral risks, such as radiological or radionuclide imaging modalities.

Studies focusing on cost–benefit analyses have emphasized the crucial role of non-invasive imaging in determining which patients should undergo pricier diagnostic tests, such as invasive coronary procedures.^1^ Accordingly, in 2023, the Multimodality Appropriate Use Criteria (AUC) for the detection and risk assessment of coronary artery disease have been revised and published by several societies (ACC/AHA/ASE/ASNC/ASPC/HFSA/HRS/SCAI/SCCT/SCMR/STS).^2^ However, the widespread adoption of such guidelines and the reduction of unsuitable nuclear stress tests have seen only modest success. Notwithstanding these challenges, AUCs are still the recognized standard for gauging suitability in nuclear cardiology.

Our objective in this study was to scrutinize AUCs among a comprehensive group of consecutive patients who were directed to our nuclear cardiology laboratory for stress myocardial perfusion imaging (MPI). The adherence to the indication for the functional test in the diagnostic workflow, as proposed by the ‘European Society of Cardiology’ (ESC) guidelines on chronic coronary syndromes (CCSs)^3^, was also evaluated. Furthermore, we endeavoured to explore the correlation between test appropriateness patterns and the identification of ischaemia.

## Methods

We conducted a retrospective analysis on all 1870 consecutive patients (in-patients and out-patients) referred to stress–rest myocardial MPI with a cadmium zinc telluride (CZT) camera at the nuclear cardiology laboratory of the Fondazione Toscana Gabriele Monasterio between January 2021 and December 2022. These individuals underwent stress–rest MPI procedures using a dedicated cardiac camera equipped with CZT detectors, with a 1-day protocol. Most patients (89%) were referred to MPI by clinical cardiologists, whereas general practitioners accounted for the referral of the remaining 11%.

**Consent:** Our research was approved by the Institutional Ethical Committee and was conducted in strict compliance with the Declaration of Helsinki pertaining to human studies. All participating individuals provided their written consent prior to inclusion.

### AUC classification and considerations

The appropriateness of MPI examinations was determined by referencing the structured flowchart presented in the 2023 AUC guidelines.^2^ The risk assessment for asymptomatic individuals was defined according to the one proposed in the 2023 AUC.^2^ This was further stratified into low (<5%), borderline (5–7.5%), intermediate (7.5–20%), or high (>20%) risk categories. Every patient was allocated to a singular ‘clinical scenario’ as delineated in the 2023 AUC guidelines.^2^ In instances where an MPI examination could align with multiple indications of identical appropriateness levels, the indication bearing the smallest AUC numerical designation was selected.

Additionally, for symptomatic patients, the pre-test clinical likelihood of coronary artery disease (CAD) was stratified as very low (<5%), intermediate (<15%), or high (>15%)^3^, and the level of adherence of MPI to current ESC guidelines was classified.^3,4^

### Analysis of MPI

Perfusion scans were assessed according to the 17-segment left ventricular model based on a five-point scale (0 = normal, 1 = equivocal, 2 = moderate reduction, 3 = severe reduction, and 4 = absence of detectable tracer uptake) using the Corridor 4DM-SPECT software.^5^ Moderate ischaemia was defined as 4 ≥ summed difference score (SDS) < 7, while severe ischaemia was characterized by SDS ≥ 7.^6^

### Invasive coronary angiography and coronary revascularization

Information on early invasive coronary angiography (ICA) and revascularizations in the 6 months after MPI were gathered from a subset of patients (*n* = 359, 20%) who were referred to our institution for further management. Coronary stenoses with a diameter reduction of ≥50% were considered obstructive.

### Statistical analysis

Continuous variables were expressed as mean ± standard deviation, while categorical variables were presented as percentages. The *χ*^2^ test was used for comparing categorical data, and the analysis of variance was employed for comparing continuous variables. A score of *P* < 0.05 was considered statistically significant. Statistical analyses were conducted using JMP statistical software (SAS Institute Inc., version 4.0.0) and Stata software (Stata Statistical Software: Release 10; StataCorp., 2007, College Station, TX, USA).

## Results

**Figure 1:**
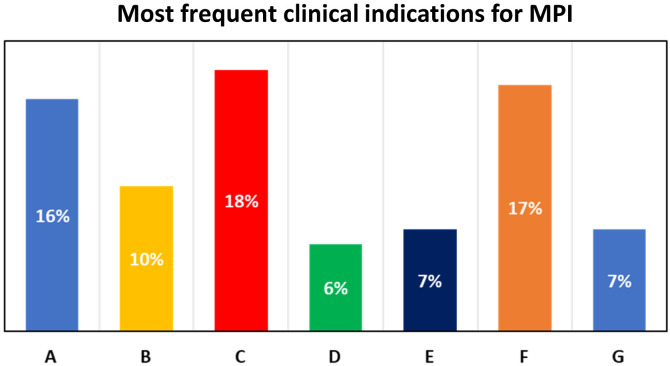
The most frequent clinical indications for myocardial perfusion imaging in the study population, according to the Multimodality Appropriate Use Criteria for the detection and risk assessment of coronary artery (ACC/AHA/ASE/ASNC/ASPC/HFSA/HRS/SCAI/SCCT/SCMR/STS),^2^ were: (A) inconclusive or abnormal exercise test (Indications 8–9; 16% of patients); (B) coronary computed tomography angiography (CCTA) inconclusive (coronary artery disease (CAD)-RADS N; Indication 17; 10% of patients); (C) prior percutaneous coronary intervention, symptoms similar to prior ischaemic episode, and/or anginal symptoms (Indication 26; 18%); (D) prior myocardial infarction (MI), no revascularization, symptoms similar to prior ischaemic episode, and/or anginal symptoms (Indication 30; 6%); (E) patients at high risk for or with a history of silent ischaemia (Indication 45; 7% of patients); (F) newly diagnosed heart failure preserved ejection fraction (HFpEF) or heart failure reduced ejection fraction (HFrEF) (Indication 51–52; 17% of patients); (G) frequent premature ventricular contractions (PVCs) or non-sustained ventricular tachycardia (Indication 55; 7% of patients).

The clinical characteristics of the 1870 enrolled patients are reported in *Table 1*. The mean age of the population was 73 ± 12 years, with 33% of patients being female. The most frequent clinical indications for MPI stress testing are reported in *Figure 1*. According to current ESC guidelines for the management of CCS, 35 (2%), 269 (14%), and 1565 (84%) patients had a low (<5%), intermediate (5–15%), and high (>15%) pre-test likelihood of CAD, respectively (*Table 2*).

**Table 1:** Characteristics of the overall population and of patients divided according to the adherence to Appropriate Use Criteria 2023 criteria

	Total (*n* = 1870)	Appropriate (*n* = 1638)	Inappropriate (*n* = 130)	Uncertain (*n* = 102)	*P*-value
Age, years	73 ± 12	73 ± 11	71 ± 15	73 ± 8	NS
Female, *n* (%)	688 (37)	598 (36)	60 (46)	30 (29)	**0.025**
Symptomatic patients with no known CAD and no prior testing					
Less-likely anginal symptoms, age <50 years and 0 or 1 CV risk factor, *n* (%)	27 (1)		27 (21)		
Less-likely anginal symptoms, age 50 years or above and/or ≥2 CV risk factors, *n* (%)	12 (0.5)			12 (12)	
Likely anginal symptoms, age <50 years and 0 or 1 CV risk factor, *n* (%)	26 (1)	26 (2)			
Likely anginal symptoms, age 50 years or above and/or ≥2 CV risk factors, *n* (%)	28 (1)	28 (2)			
Symptomatic patients without known CAD and with prior testing					
Inconclusive or abnormal exercise test, *n* (%)	298 (16)	298 (18)			
CCTA inconclusive (CAD-RADS N), *n* (%)	193 (10)	193 (12)			
CCTA with no CAD or up to 49% stenosis (CAD-RADS 0–2), *n* (%)	24 (1)			24 (24)	
CCTA with stenosis >70% (CAD-RADS 4–5), *n* (%)					
Mild ischaemia on stress imaging, *n* (%)	37 (2)		37 (28)		
Symptomatic patients with prior MI or revascularization					
Incomplete revascularization, *n* (%)	85 (5)	85 (5)			
Prior PCI, symptoms similar to prior ischaemic episode and/or anginal symptoms, *n* (%)	331 (18)	331 (20)			
Prior MI, no revascularization, symptoms similar to prior ischaemic episode and/or anginal, *n* (%)	114 (6)	114 (7)			
Prior PCI, non-anginal symptoms, *n* (%)					
Asymptomatic patients without known ASCVD					
Intermediate atherosclerotic cardiovascular risk 7.5–20% with or without risk-enhancing factors, *n* (%)	56 (3)		56 (43)		
High atherosclerotic cardiovascular disease risk >20%, *n* (%)	6 (0.3)			6 (6)	
Asymptomatic patients with prior revascularization or MI					
Patients at high risk for or with a history of silent ischaemia, *n* (%)	123 (7)	123 (8)			
Incomplete revascularization, *n* (%)	20 (1)			20 (20)	
>5 years after CABG, *n* (%)	10 (0.5)			10 (10)	
<2 years after PCI, *n* (%)	10 (0.5)		10 (8)		
>2 years after PCI, *n* (%)	30 (2)			30 (29)	
Other cardiovascular conditions in patients without symptoms of ischaemia					
Newly diagnosed HFpEF or HFrEF, *n* (%)	316 (17)	316 (19)			
Frequent PVCs or non-sustained VT, *n* (%)	124 (7)	124 (8)			
Myocardial perfusion imaging					
SDS < 4, *n* (%)	333 (18)	143 (9)	120 (92)	70 (69)	**<0.001**
4 ≤ SDS < 7, *n* (%)	612 (33)	594 (36)	10 (8)	8 (8)	
SDS ≥ 7, *n* (%)	926 (50)	902 (55)	0	24 (23)	

CAD, coronary artery disease; CCTA, coronary computed tomography angiography; CV, CardioVascular; HFpEF, heart failure preserved ejection fraction; HFrEF, heart failure reduced ejection fraction; MI, myocardial infarction; NS, not significant; PVC, premature ventricular contractions; SDS, summed difference score.

**Table 2:** Characteristics of patients divided according to the adherence to ESC guidelines for the management of ‘chronic coronary syndromes’

	Adherent (*n* = 1685)	Non-adherent (*n* = 140)	Uncertain (*n* = 45)	*P*-value
Age, years	73 ± 11	71 ± 15	73 ± 8	NS
Female, *n* (%)	620 (37)	46 (33)	17 (38)	**0**.**022**
Symptoms				
Typical angina, *n* (%)	784 (47)	0	0	**<0**.**001**
Atypical angina, *n* (%)	294 (17)	0	12 (27)	
Non-cardiac chest pain, *n* (%)	100 (6)	36 (23)	0	
Dyspnoea, *n* (%)	354 (21)	40 (29)	0	
Asymptomatic, *n* (%)	156 (9)	66 (47)	33 (73)	
Pre-test likelihood of CAD				
<5%, *n* (%)	0	35 (25)	0	**<0**.**001**
5–15%, *n* (%)	227 (13)	35 (25)	7 (18)	
>15%, *n* (%)	1458 (86)	70 (50)	37 (82)	
Myocardial perfusion imaging				
SDS < 4, *n* (%)	170 (10)	120 (86)	39 (87)	**<0**.**001**
4 ≤ SDS < 7, *n* (%)	594 (35)	20 (14)	1 (2)	
SDS ≥ 7, *n* (%)	920 (55)	0	5 (11)	
Coronary anatomy (359 patients)				
Obstructive CAD (≥50%), *n* (%)	278 (93)	17 (49)	11 (44)	**<0**.**001**
No CAD/non-obstructive CAD, *n* (%)	21 (7)	18 (51)	14 (56)	
Coronary revascularization, *n* (%)	193 (65)	7 (20)	7 (28)	**<0**.**001**

NS, not significant; SDS, summed difference score.

### Evaluation of test appropriateness

According to the AUC criteria, the majority of MPI stress tests performed were classified as clinically appropriate (1638 patients, 88%), while 102 (5%) were categorized as uncertain appropriateness, and 130 (7%) were deemed clearly inappropriate (*Table 1*).

The evaluation of the adherence of MPI to ESC guidelines for the management of CCS is represented in *Table 2*. While in every patient with a low pre-test likelihood of CAD, the use of MPI was non-adherent to guidelines, this proportion dropped to 9 and 4% (*P* < 0.05 for trend) in patients with intermediate and high pre-test likelihood, respectively.

### Interplay between appropriateness and MPI results

We examined the relationship between the appropriateness of stress tests and the presence of ischaemia on MPI.

While the majority of clinically appropriate MPI tests revealed the presence of moderate-to-severe ischaemia, only a small proportion of stress tests with uncertain clinical appropriateness or clearly inappropriate indications showed substantial myocardial ischaemic burden (*Table 1*).

When adherence to ESC guidelines was respected, the majority (55%) of the patients showed moderate-to-severe ischaemia, a condition that was present in none of the patients managed non-adherently to guidelines (*P* < 0.05; *Table 2*).

### Adherence to ESC guidelines and early follow-up

A significantly higher rate of obstructive CAD (93 vs. 47%, *P* < 0.001) and early coronary revascularization (65 vs. 23%, *P* < 0.001) following MPI was demonstrated in patients managed adherently to ESC guidelines than in those managed non-adherently (*Table 2*).

## Discussion

The present results, obtained in a referral centre for nuclear cardiology, underscore that the majority of clinical indications for MPI stress tests aligns with AUC criteria. Moreover, despite a marked variance in test-ordering practices compared with previously published data, appropriate MPI tests showed a high diagnostic yield and possibly better downstream resource utilization compared with inappropriate ones.

According to available data, combining a deep understanding of clinical evidence with a technical knowledge of imaging modalities facilitates appropriate test indications, reducing healthcare costs and patients’ overall risk burden.^2,6,7^

In our laboratory, the presence of a dedicated specialist in nuclear cardiology has contributed to improved service quality from both technical and clinical perspectives. This competence enhances clinical appropriateness for imaging studies, benefiting referring physicians and nuclear cardiologists.

This approach aligns with the strategy of the European Association of Cardiovascular Imaging, which offers individual certification and laboratory accreditation programmes to enhance professional excellence across Europe, standardizing practice quality, improving accuracy, and reducing costs.^8^

The second key aspect of this study is the clinical impact of adhering to AUC.^2^ When a test indication aligns with ESC guidelines or AUC criteria,^2,3^ one can anticipate an improvement of its diagnostic yield, as demonstrated by a significantly higher prevalence of MPI tests showing moderate-to-severe ischaemia than in patients managed non-adherently (55 vs. 0%), identifying the likely benefit of coronary revascularization. Accordingly, in patients managed in adherence to ESC guidelines, ICA more frequently showed obstructive CAD leading to coronary revascularization than in those managed non-adherently. These results are in line with recent evidence demonstrating that approaches ensuring the appropriateness of MPI interpretation and adherence to guidelines may guide the referral for coronary angiography and revascularization and be associated with an improved prognosis.^9,10^ The rate of patients managed adherently to current guidelines in the present paper is higher than that reported in a recent pan-European registry^4^ (90 vs. 56% in the EURECA registry), a factor that might be related to the single-centre, single-national, single-modality nature of our study and that may need confirmation in a wider, multinational, population.

Only a limited fraction of studies with uncertain clinical appropriateness or clearly inappropriate indications revealed a significant ischaemic burden or obstructive CAD. These latter categories mainly identified patients at lower cardiovascular risk, such as asymptomatic individuals with a low pre-test probability of CAD. Applying AUC criteria in routine practice can help reduce unnecessary testing, focusing clinical efforts on intermediate-risk patients in whom test results are more likely to impact cardiological management and overall clinical outcomes. This approach may decrease healthcare costs and the need for high-risk investigations such as invasive coronary procedures.

### Limitations

This is a single-centre study, potentially introducing some degree of selection bias. Despite being retrospective, we consecutively enrolled all patients undergoing MPI in a large time frame, minimizing selection bias. Additionally, since most MPI tests were referred by clinical cardiologists, the observed level of appropriateness may be artificially elevated. Finally, data on the referral to ICA and coronary revascularization were available only in a limited proportion of patients, making such analysis only exploratory.

## Conclusion

The rate of inappropriate MPI tests is notably low in a high-volume laboratory. Appropriate and inappropriate studies identified patients at high and low probabilities of significant ischaemia and predicted better downstream resource utilization, respectively.

## Data Availability

The data underlying this article will be shared on reasonable request to the corresponding author.

## Lead author biography

Alessia Gimelli is a mid-career female cardiologist and nuclear medicine specialist with a specific interest in cardiac imaging. She has multiple senior roles both in her home institution and in several scientific and professional societies. She is the clinical lead of the Nuclear Cardiology unit, FTGM, and she has been leading the Nuclear Cardiology clinical training programme since 2012 at the University of Pisa, Italy. She leads the health quality system programme of the FTGM. She has participated in many clinical research projects and authored numerous peer-reviewed articles. She was the treasurer of the European Association of Cardiovascular Imaging (EACVI) between 2020 and 2022) and EACVI vice president (Chair of Nuclear Cardiology and CT section 2018–20). She is the Editor in Chief of the *EHJ Imaging Methods and Practice*.
